# Posterior Capsule Folds as a Potential Early Sign of Positive Vitreous Pressure During Cataract Surgery: A Case Series

**DOI:** 10.7759/cureus.113047

**Published:** 2026-07-20

**Authors:** Wenwen Gong, Cooper Bahr, Haley Peterson, Gabriella Dolinsky, Christopher Shelby, Wyche T Coleman, Stephen LoBue

**Affiliations:** 1 Department of Ophthalmology, Willis-Knighton Medical Center, Shreveport, USA; 2 College of Medicine, Drexel University, Philadelphia, USA; 3 School of Medicine, St. George's University, St. George’s, GRD; 4 Department of Ophthalmology, Louisiana State University Health Sciences Center, Shreveport, USA

**Keywords:** cataract surgery, choroidal effusion, fluid misdirection syndrome, phacoemulsification cataract surgery, positive vitreous pressure

## Abstract

Positive vitreous pressure (PVP) is a rare phenomenon encountered in intraocular surgery where increased vitreous pressure pushes the lens-iris diaphragm forward, which can result in posterior capsule bulging, shallowing of the anterior chamber (AC), iris prolapse, and intraocular lens (IOL) malposition. We present four cases of early PVP that presented as posterior capsule folds or wrinkles during cataract surgery or combined cataract surgery with minimally invasive glaucoma surgery (MIGS). Dense nuclear sclerosis and generalized zonular weakness were present in three of the four cases. In all cases, subtle capsular folds were seen during or shortly after IOL insertion. In all cases, AC shallowing or tilting of the IOL out of the capsular bag was seen. In three cases where early recognition of PVP was performed, vitreous pressure was successfully managed with wound closure and adjunctive intravenous mannitol or systemic acetazolamide with good visual outcomes of 20/25 or better. Late recognition in one case resulted in a posterior capsular rupture with vitreous loss from a silicone IOL puncturing the bag. Overall, early recognition of PVP, which may be associated with posterior capsule folds or wrinkles, can be helpful for anterior segment surgeons mitigating complications or invasive surgical treatments.

## Introduction

Positive vitreous pressure (PVP) is a rare phenomenon encountered in intraocular anterior segment surgery and may lead to severe complications if not recognized and managed promptly [1]. PVP develops when the pressure gradient between the posterior segment and the anterior segment increases, pushing the lens-iris diaphragm forward and resulting in posterior capsule bulging, shallowing of the anterior chamber (AC), iris prolapse, and in severe cases, posterior capsular rupture (PCR) and vitreous loss [1]. Eyes with short axial length, shallow AC, dense cataracts, or zonulopathy are at higher risk [2,3].

Although the classic signs of PVP, including a rock-hard eye [2], severe AC shallowing that is unable to reform, and iris prolapse, are well recognized, subtle early findings may be overlooked. Early recognition and intervention are important as they can lead to better outcomes with less invasive surgical techniques and treatment.

We present a case series of four patients in which posterior capsular folds or wrinkles were the early sign of PVP during cataract surgery or combined cataract surgery with minimally invasive glaucoma surgery (MIGS). In three cases, early recognition allowed prompt wound closure and medical management without further complications. To our knowledge, based on our review of the available literature, this potential sign of early PVP has not been clearly reported.

## Case presentation

Case 1

A 75-year-old man with a medical history of prediabetes mellitus, hypertension, and atrial fibrillation presented for evaluation for decreased vision in both eyes (OU). He had no previous history of ocular surgery. Best corrected visual acuity (BCVA) was 20/60 in the right eye (OD) and 20/40-1 in the left eye (OS). Slit lamp examination (SLE) was notable for 3.5+ and 3+ nuclear sclerosis (NS) OD and OS respectively. The dilated fundus exam was unremarkable. The patient underwent femtosecond-assisted cataract surgery with a monofocal lens OU. Cataract surgery was staged with the right eye proceeding first.

**Figure 1:**
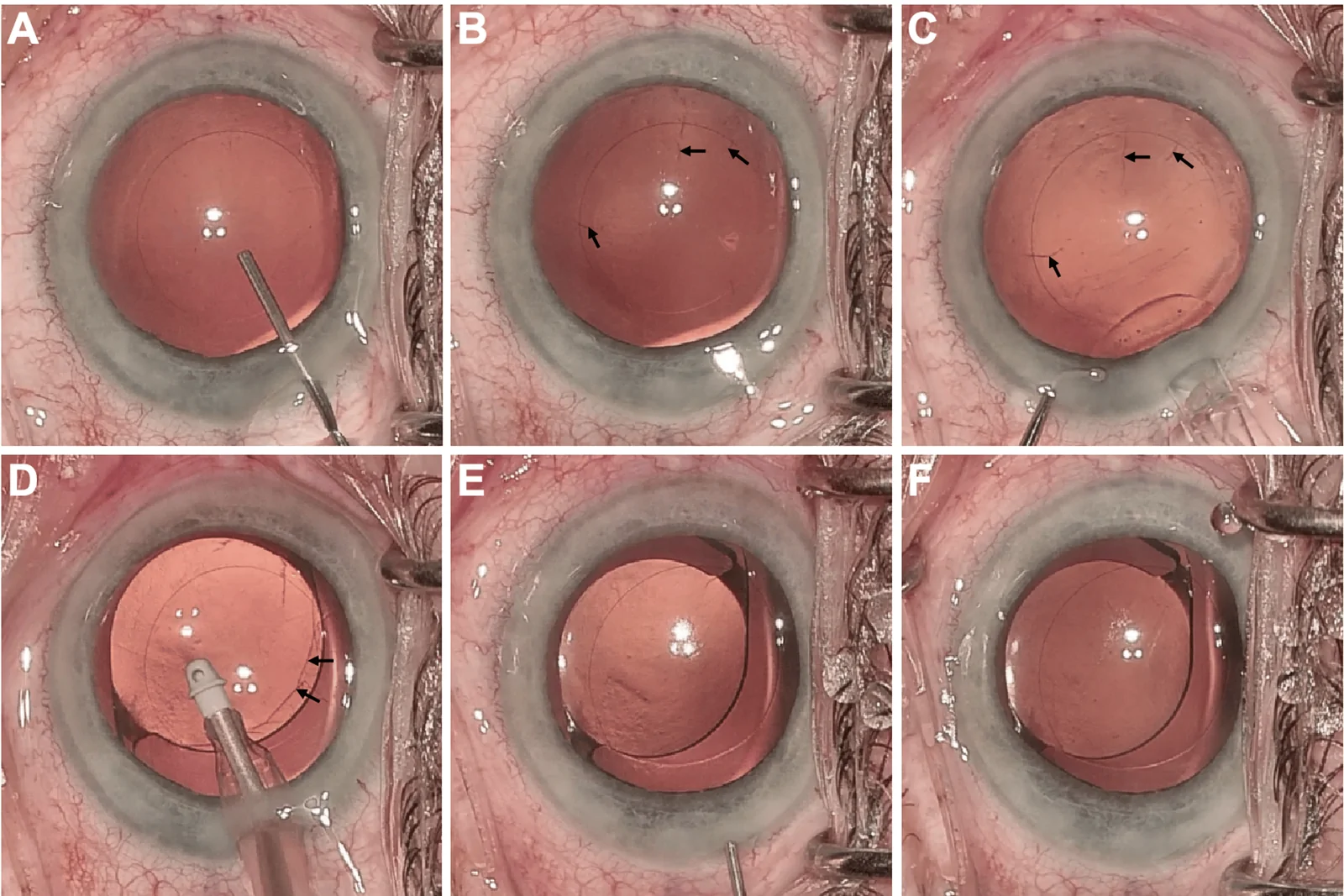
Case 1 Before Acetazolamide. (A) After removal of the nucleus and cortex, an ophthalmic viscoelastic device was injected into the capsular bag, and no obvious posterior capsule (PC) folds/wrinkles were present. (B) Before intraocular lens (IOL) insertion, PC folds/wrinkles appeared (black arrows), representing an early sign of positive vitreous pressure. (C) During IOL insertion, the PC folds/wrinkles (black arrows) became deeper and more prominent. (D-E) Unable to position the IOL within the capsular bag, black arrows indicating PC folds/winkles. (F) No improvement after loosening the lid speculum.

**Figure 2:**
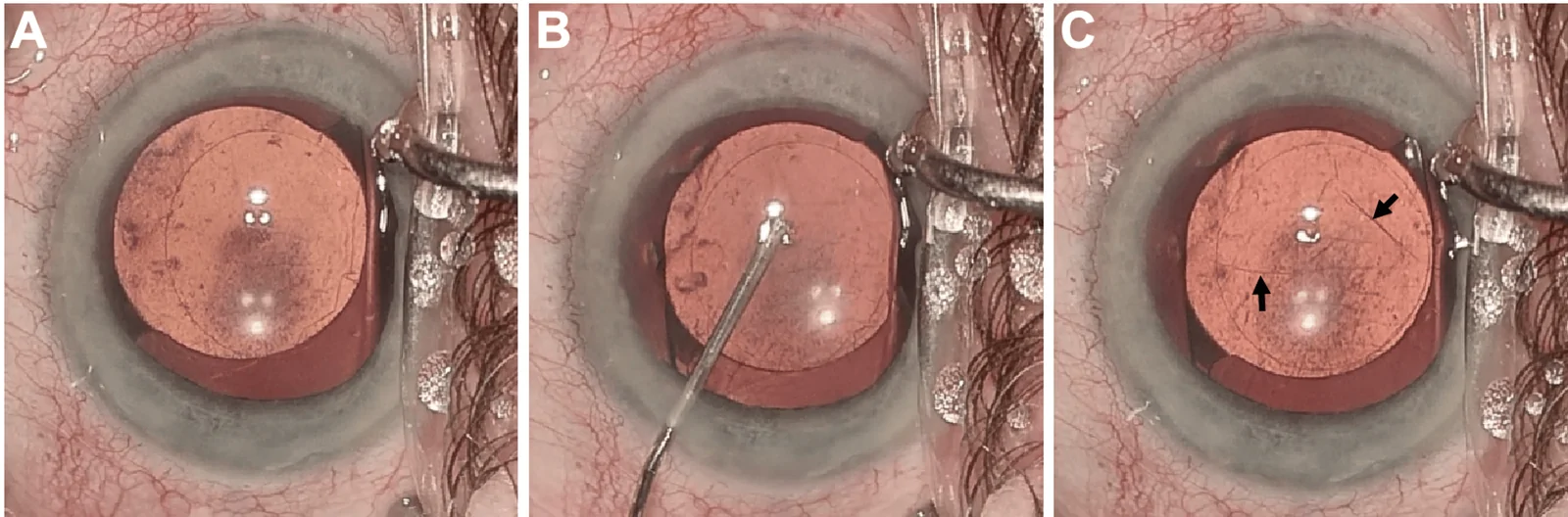
Case 1 After Acetazolamide. (A-B) Intraocular lens (IOL) successfully repositioned into the capsular bag two hours after intravenous administration of 500 mg acetazolamide. (C) Black arrows highlighting the capsular folds after intravenous acetazolamide (positive vitreous pressure has decreased but is still present).

Moderate global zonular laxity was noted intraoperatively OD during phacoemulsification and cortex removal. Following cortex removal, shallowing of the AC was noted. After placement of cohesive ophthalmic viscoelastic device (OVD), mild posterior capsular wrinkling was initially observed, followed by progressive posterior pressure, further shallowing of the AC, and difficulty positioning the intraocular lens (IOL) within the capsular bag (Figure 1). The eye was firm but not hard. No loss of red-reflex was observed. Relaxing the lid speculum did not improve the posterior pressure, suggesting that external compression was unlikely to be the primary mechanism. The eye was closed with a suture and 500 mg of intravenous acetazolamide was administered. Approximately two hours later, the IOL was successfully repositioned into the capsular bag and the case was completed without further complications (Figure 2). Postoperatively the patient did well with no complications and an uncorrected visual acuity (VA) of 20/20- OD.

Case 2

A 73-year-old man with diabetes mellitus, hypertension, and no history of prior ocular surgery presented to clinic due to worsening nighttime glare OU. BCVA was 20/25 OU with brightness acuity test (BAT) of 20/40-2 OU. SLE was notable for 2+ NS OU. The dilated fundus exam was unremarkable. The patient underwent standard cataract surgery with a monofocal lens OU. Cataract surgery was staged with the left eye first.

**Figure 3:**
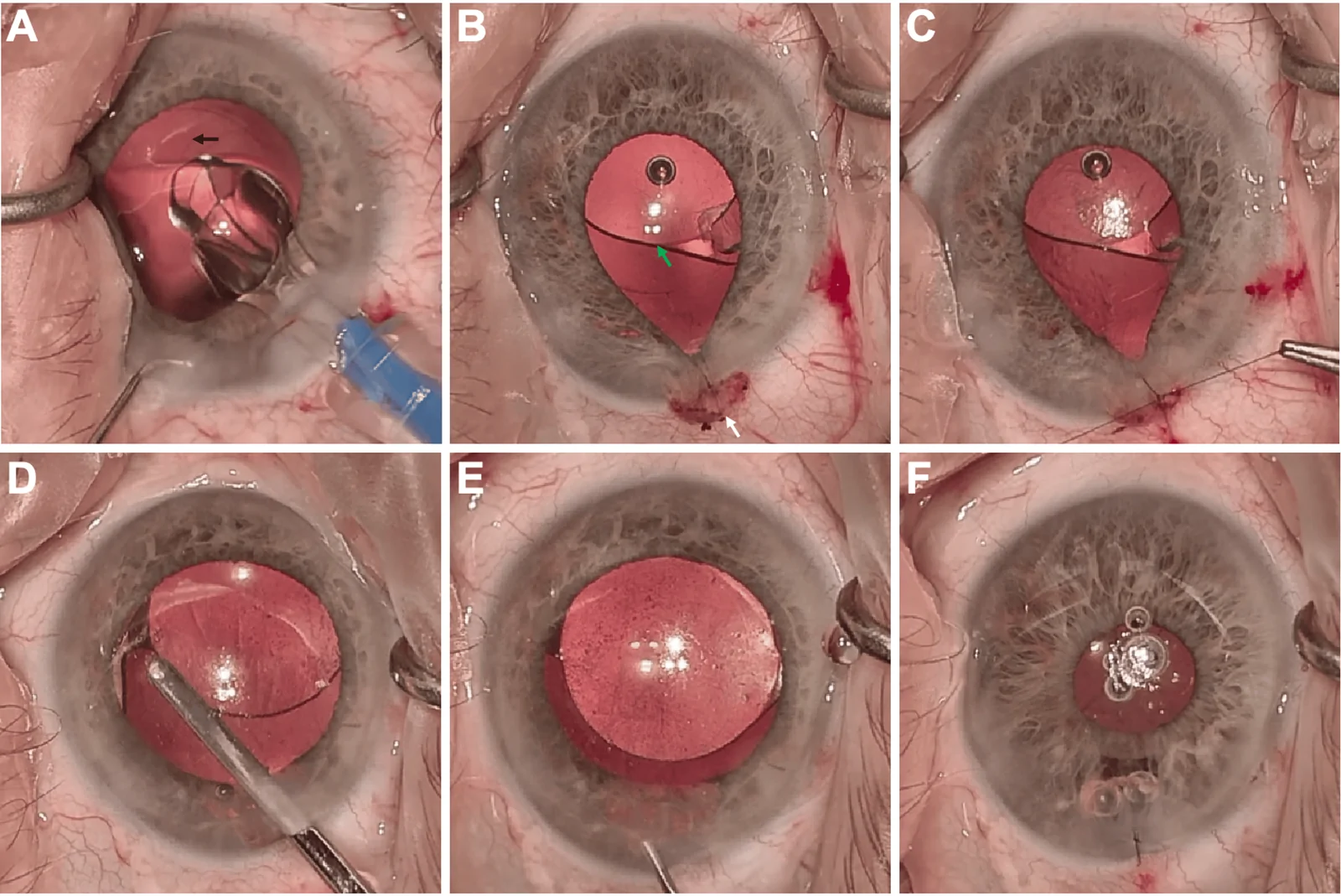
Case 2. (A) Capsular folds/wrinkles (black arrow) during intraocular lens (IOL) insertion. (B) Iris prolapse (white arrow), IOL tilted and not positioned within capsular bag (green arrow). (C) Wound was closed with a suture. (D-E) Two hours after intravenous mannitol, IOL repositioned into the capsular bag. (F) Miosis induced and wound sutured.

During IOL insertion, the AC shallowed significantly due to a tight wound. Mild posterior capsular folds were noted at this time (Figure 3A). Progressive shallowing of the AC was noted with the IOL tilting out of the capsular bag and iris prolapse (Figure 3B).

The eye was firm but not hard with no loss of the red-reflex. The wound was sutured to close the eye (Figure 3C). Next, intravenous 25% mannitol was administered at 1 g/kg body weight. Approximately two hours later, the IOL was repositioned successfully into the capsular bag (Figure 3D, 3E). Intracameral carbachol 0.01% 0.5 mL was administered to constrict the pupil. A suture was placed to close the wound, and the remainder of the surgery was completed uneventfully (Figure 3F). Postoperatively the patient did well with no complications and an uncorrected VA of 20/20 OS.

Case 3

A 70-year-old man with mild primary open angle glaucoma and no medical history presented due to significant vision loss in OD. BCVA was 20/500 OD and 20/60 OS. SLE was notable for pseudoexfoliated material on the anterior lens capsule, 3mm pupil, and 3.5+ NS. The dilated fundus exam was grossly normal. Ultrasound also confirmed no gross abnormalities. The patient was planned for staged cataract surgery with MIGS OU with a monofocal IOL. The right eye proceeded first with surgery.

**Figure 4:**
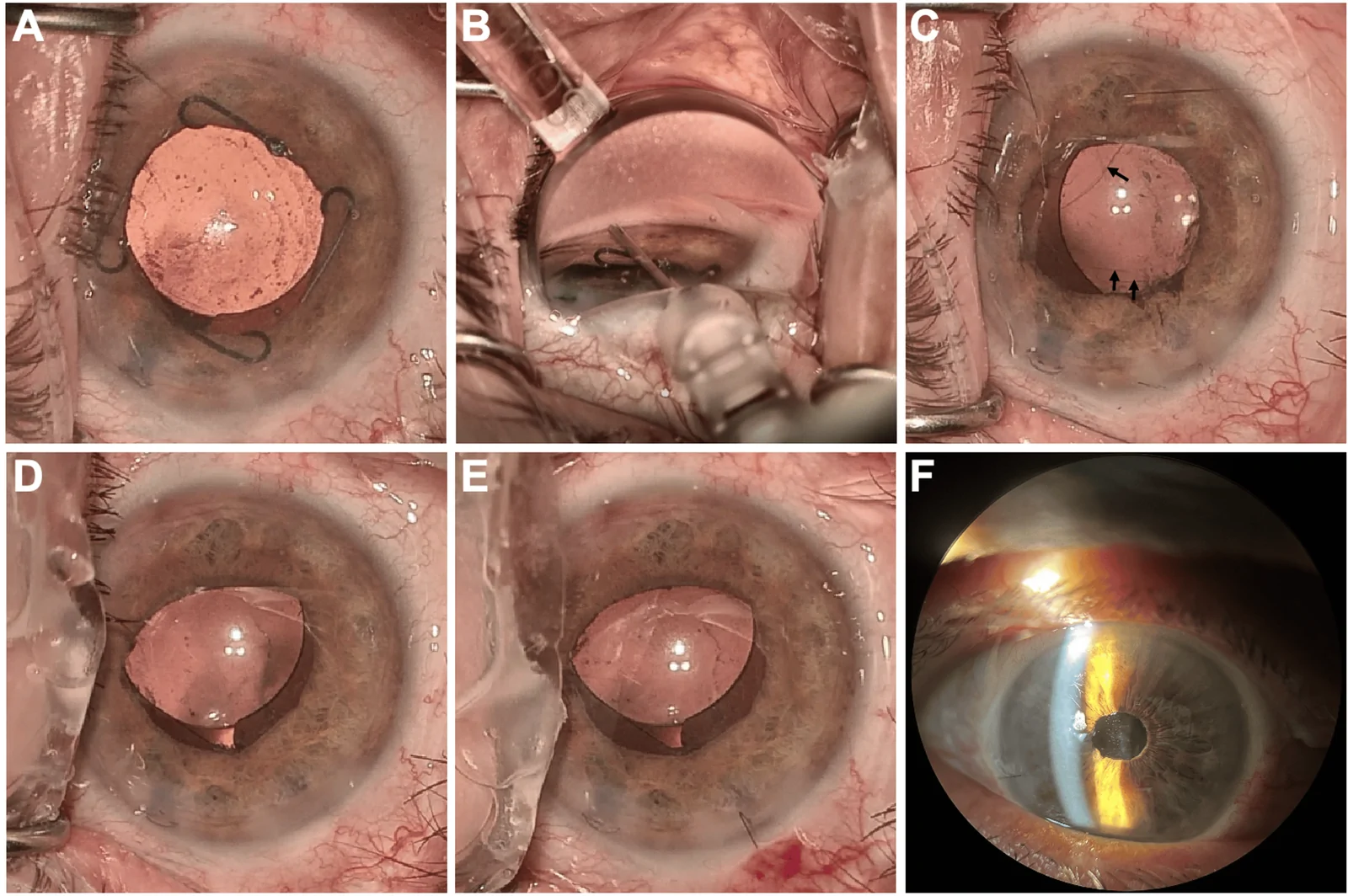
Case 3. (A) Intraocular lens (IOL) centered within the capsular bag. (B) Three trabecular meshwork (TM) stents were placed in the nasal TM. (C) Capsular folds/wrinkles (black arrows) were noted. (D) IOL displaced from the capsular bag and unable to be repositioned. (E) A suture was placed to close the wound, 1 g oral acetazolamide was given postoperatively. (F) IOL in good position on postoperative day 1.

A Malyugin ring (MicroSurgical Technology, Redmond, WA, USA) was placed to expand the pupil, and trypan blue was used to stain the anterior capsule. Moderate global zonular laxity was noted during nucleus and cortex removal. The IOL was initially placed within the capsular bag with good positioning (Figure 4A). Three trabecular micro-bypass stents (iStent infinite Trabecular Micro-Bypass System Model iS3; Glaukos Corporation, San Clemente, CA, USA) were then placed in the nasal trabecular meshwork (Figure 4B). The AC shallowed after stent placement due to loss of OVD from the main wound. Shortly after, posterior capsular wrinkles and folds were noted (Figure 4C) with the IOL tilting out of the capsular bag with inability to reposition (Figure 4D). The eye was firm but not hard with no loss of the red-reflex. The wound was closed with a suture, and 1 g of oral acetazolamide was given postoperatively (Figure 4E). On postoperative day 1, the IOL had spontaneously settled into the capsular bag and was in good position, with a round pupil and normal intraocular pressure (Figure 4F). The postoperative period was uncomplicated with uncorrected VA of 20/25+ by one week postoperatively.

Case 4

An 82-year-old man with Parkinson disease, mild primary open-angle glaucoma OU, and intermediate age-related macular degeneration, presented to the clinic due to decreased vision OU. BCVA was 20/50 OD and 20/80 OS. SLE was notable for 3+ and 3.5+ NS OD and OS respectively. A dilated fundus exam was unremarkable. The patient underwent cataract surgery with MIGS and the light adjustable lens (LAL) (Rx Sight, Aliso Viejo, CA, USA) OU. Surgery proceeded first for the left eye.

**Figure 5:**
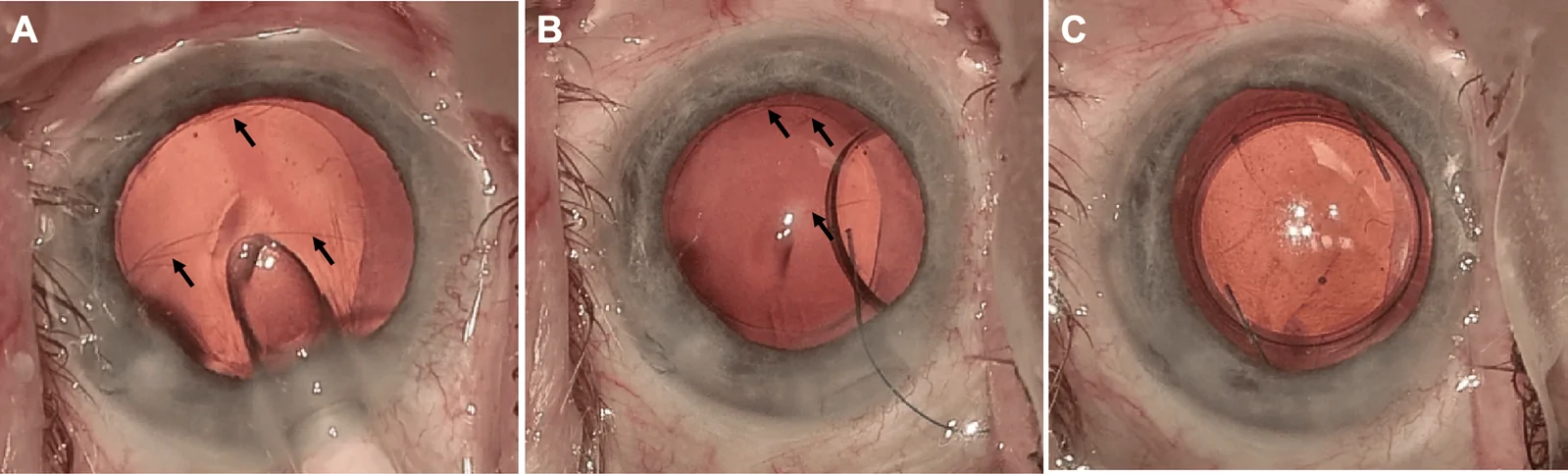
Case 4. (A) Prominent posterior capsular wrinkles/folds (black arrows) were noted during insertion of the light adjustable lens (LAL). (B) Posterior capsular wrinkles/folds (black arrows) persisted, and the LAL displaced posteriorly through the posterior capsule, resulting in posterior capsular rupture. (C) LAL in the ciliary sulcus.

Trypan blue was used to stain the anterior capsule. Moderate global zonulopathy was noted during phacoemulsification and cortex aspiration. During cohesive OVD placement prior to the LAL insertion, the AC was shallow with prominent posterior capsular wrinkles and folds (Figure 5A). The explosive nature of the silicone LAL ruptured the posterior capsule with the haptics and allowed vitreous to prolapse through the main wound (Figure 5B). An anterior vitrectomy was performed, and the LAL was placed in the ciliary sulcus (Figure 5C). Intracameral carbachol 0.01% 0.5 mL was used to constrict the pupil, and the wound was closed with a 10-0 nylon suture. Postoperative fundus photography and macular optical coherence tomography (OCT) demonstrated nasal and temporal chorioretinal folds outside the foveal, suggesting that intraoperative choroidal effusion may have contributed to the development of intraoperative PVP. The patient was referred to retina. No holes or tears were noted. The chorioretinal folds resolved spontaneously over two months. Uncorrected VA was 20/25 after three months once the LAL was adjusted and locked in.

Baseline age, sex, medical history, ocular history, and biometric characteristics of the four cases are summarized in Table 1.

**Table 1:** Baseline Characteristics Abbreviations: M, male; Hx, history; DM, diabetes mellitus; HTN, hypertension; A-fib, atrial fibrillation; PD, Parkinson disease; PXF, pseudoexfoliative syndrome; POAG, primary open-angle glaucoma; i-AMD, intermediate age-related macular degeneration; OU, both eyes; AL, axial length; ACD, anterior chamber depth; LT, lens thickness; NS, nuclear sclerosis.

Case	Age, years	Sex	Medical Hx	Ocular Hx	AL, mm	ACD, mm	LT, mm	NS grade	Zonular laxity
1	75	M	Pre-DM, HTN, A-fib	None	23.39	2.55	5.86	3.5+	Moderate
2	73	M	DM, HTN	None	23.48	3.05	4.93	2+	None
3	70	M	None	PXF	23.70	3.42	5.11	3.5+	Moderate
4	82	M	PD	Mild POAG OU, i-AMD OU	23.28	2.81	5.09	3.5+	Moderate

## Discussion

Our case series presents posterior capsular folds or wrinkles as a potential early sign of PVP during anterior segment surgery. We hypothesize that the posterior capsular folds are caused by directed pressure on the posterior capsule from retrocapsular fluid or the anterior vitreous, which leads to wrinkles and shallowing of the AC.

In cases 1, 2, and 4, posterior capsular wrinkling occurred before or during IOL insertion, whereas in case 3, it occurred after placement of the trabecular micro-bypass stents. In cases 1-3, capsular wrinkling was accompanied by difficulty positioning the IOL within the capsular bag. AC shallowing occurred without loss of a red-reflex occurred in every case. Dense NS and moderate zonular laxity were also seen in 75% of the cases.

Three cases were managed successfully and non-invasively with good outcomes when PVP was recognized early. Acetazolamide or mannitol were used to deepen the AC and reposition the IOL which was tilted out of the capsular bag in cases 1-3. Again, capsular wrinkles during or after IOL insertion proceeded progressively worsening PVP in all cases and may be an early sign of vitreous pressure.

PCR occurred in one case due to the late recognition of PVP. The explosive nature of LAL in a shallow AC with increased vitreous pressure led to the haptics puncturing the posterior capsule with subsequent vitreous loss. PCR and vitreous loss could have been prevented if the case was stopped with IOL insertion performed at a later time or if the wound was closed and the patient was treated with acetazolamide or mannitol to reduce PVP and create more room for IOL insertion.

The mechanism of PVP has been documented to occur from acute intraoperative hypotony, increased posterior pressure caused by pressure on the sclera, choroidal effusion or suprachoroidal hemorrhage, or acute fluid misdirection syndrome [1]. Acute fluid misdirection syndrome occurs when irrigating fluid enters the Berger’s space through the zonules [4]. Irrigating fluid may also enter the vitreous cavity if there is a tear in the anterior hyaloid membrane [5]. In cases 1-3, no scleral compression, choroidal effusion, or suprachoroidal hemorrhage was observed. Therefore, we believe that the underlying mechanism in cases 1 and 2 was acute fluid misdirection syndrome. On the other hand, case 3 was likely related to acute hypotony after placement of three trabecular micro-bypass stents, with possible contribution from acute fluid misdirection syndrome. In case 4, PVP was likely due to choroidal effusion, which was observed on imaging in the postoperative period.

Risk factors for fluid misdirection syndrome include shallow AC, short axial length [2], and zonular defects associated with primary angle-closure disease, trauma, brunescent cataract, or pseudoexfoliative syndrome [3,6]. In our series, zonular laxity and dense NS were present in cases 1, 3, and 4 and may have contributed to the development of acute fluid misdirection syndrome.

Our cases suggest that posterior capsular folds or wrinkles should prompt the surgeon to assess for early posterior pressure. Recognition of this sign may be particularly important in eyes with risk factors such as pseudoexfoliative syndrome, zonulopathy, short axial length, shallow AC, dense cataract, or combined cataract surgery with MIGS. However, posterior capsular folds associated with PVP should be distinguished from posterior capsular folds or striae during routine cataract surgery. Posterior capsular folds or striae during routine cataract surgery typically occur after the IOL has been placed within the capsular bag and the OVD has been aspirated, and are generally linear, extending along the IOL’s maximum diameter, where the capsular bag is under the greatest tension [7]. In three of our four cases, however, the folds appeared after OVD was injected into the capsular bag but before IOL implantation. They were also associated with AC shallowing and difficulty positioning the IOL within the capsular bag. Posterior capsular folds associated with PVP may be accompanied by other manifestations of increased posterior pressure, including posterior capsular bulging and rupture, iris prolapse, and vitreous loss. In selected mild cases of PVP, early recognition followed by wound closure and adjunctive medical therapy (e.g. acetazolamide or mannitol) may be sufficient without the need for additional surgical intervention. Reports on the use of acetazolamide or mannitol for acute fluid misdirection syndrome are limited in the English-language literature. In three cases in which PVP was recognized early, vitreous pressure was successfully managed with systemic acetazolamide or mannitol. However, the independent effects of these medications cannot be determined because irrigation was discontinued, the wound was closed, and time was allowed for the misdirected fluid to redistribute or be absorbed, which may also have contributed to the resolution of PVP. It is also important to note that these medications should not be used alone to treat severe cases of PVP secondary to suprachoroidal hemorrhage or severe acute fluid misdirection. In cases of acute fluid misdirection where the globe is rock-hard, surgical decompression by pars plana needle aspiration of retrocapsular fluid [2,8] or irido-zonulo-hyaloido-vitrectomy through the main corneal incision [9] has been reported to be an effective treatment.

The distinction between acetazolamide and mannitol is important. Mannitol is a hyperosmotic agent that acts by increasing plasma osmolarity and creating an osmotic gradient between the intravascular space and the vitreous, thereby drawing fluid from the vitreous into the circulation [10]. Acetazolamide is an aqueous suppressant and by reducing aqueous production, it may decrease ongoing aqueous inflow into the posterior segment and decrease posterior pressure [11].

This case series has several limitations. First, because the sample size was small, the generalizability of these findings is limited. Second, there was no control group, so the true therapeutic effect of acetazolamide or mannitol could not be determined. Finally, acetazolamide and mannitol have delayed onset and may not be appropriate as primary treatment in severe or rapidly progressive PVP. Further studies with larger sample sizes and control groups are needed to confirm these findings.

## Conclusions

PVP is a rare phenomenon encountered in intraocular surgery where increased vitreous pressure pushes the lens-iris diaphragm forward, which can result in posterior capsule bulging, shallowing of the AC, iris prolapse, and IOL malposition. Posterior capsular folds or wrinkles may represent a potential early sign of intraoperative PVP during intraocular surgery. Early recognition of this subtle finding may allow timely intervention to prevent severe complications including PCR and vitreous loss. In selected mild cases without systemic contraindications, wound closure and adjunctive treatment with acetazolamide or mannitol may restore the equilibrium between the anterior and posterior segments and avoid more invasive surgical intervention. However, severe PVP should be managed surgically when indicated.
